# Selective Emission Fabric for Indoor and Outdoor Passive Radiative Cooling in Personal Thermal Management

**DOI:** 10.1007/s40820-025-01713-4

**Published:** 2025-03-19

**Authors:** Haijiao Yu, Jiqing Lu, Jie Yan, Tian Bai, Zhaoxuan Niu, Bin Ye, Wanli Cheng, Dong Wang, Siqi Huan, Guangping Han

**Affiliations:** 1 Key Laboratory of Bio-Based Material Science and Technology (Northeast Forestry University), Ministry of Education, Harbin, 150040 People’s Republic of China; 2 Department of Astronautical Science and Mechanics, Harbin Institute of Technology (HIT), Harbin, 150001 People’s Republic of China

**Keywords:** Passive radiative cooling, Electrospinning, Full-scale structure, Selective emission, Personal thermal management

## Abstract

Radiative cooling fabric creates a thermally comfortable environment without energy input, providing a sustainable approach to personal thermal management. However, most currently reported fabrics mainly focus on outdoor cooling, ignoring to achieve simultaneous cooling both indoors and outdoors, thereby weakening the overall cooling performance. Herein, a full-scale structure fabric with selective emission properties is constructed for simultaneous indoor and outdoor cooling. The fabric achieves 94% reflectance performance in the sunlight band (0.3–2.5 µm) and 6% in the mid-infrared band (2.5–25 µm), effectively minimizing heat absorption and radiation release obstruction. It also demonstrates 81% radiative emission performance in the atmospheric window band (8–13 µm) and 25% radiative transmission performance in the mid-infrared band (2.5–25 μm), providing 60 and 26 W m^−2^ net cooling power outdoors and indoors. In practical applications, the fabric achieves excellent indoor and outdoor human cooling, with temperatures 1.4–5.5 °C lower than typical polydimethylsiloxane film. This work proposes a novel design for the advanced radiative cooling fabric, offering significant potential to realize sustainable personal thermal management.

**Supplementary Information:**

The online version contains supplementary material available at 10.1007/s40820-025-01713-4.

## Highlights

- Full-scale structure fabric across 0.3-25 μm was fabricated for enhanced sunlight reflectance (94%) and reduced mid-infrared reflectance (6%).
- Transmission (25%) and emission (81%) characteristics for thermal radiation release.
- Indoor and outdoor cooling fabric in personal thermal management.

**Supplementary Information:**

The online version contains supplementary material available at 10.1007/s40820-025-01713-4.

## Graphical abstract

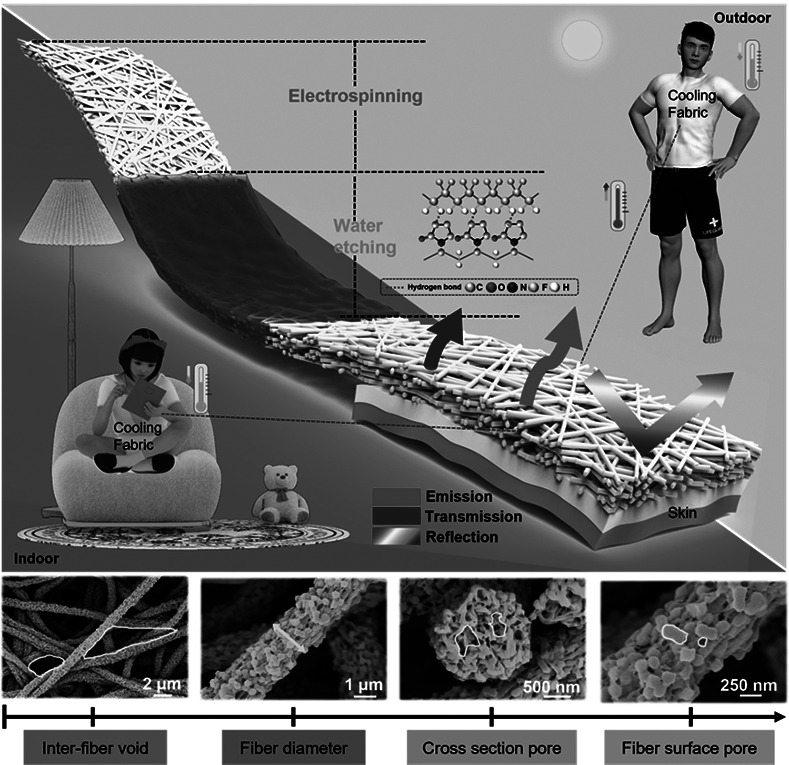

## Introduction

The greenhouse effect has significantly contributed to the increased frequency and intensity of extreme weather events [1–3]. Statistics indicate that people have endured 86 days of high-temperature weather annually over the past three years [4]. Energy-intensive refrigeration systems like air conditioners and fans are widely used to lower indoor temperatures, while they also further exacerbate the greenhouse effect [5–7]. In contrast, personal thermal management (PTM) technology has gained significant attention for its superior energy efficiency, providing localized cooling to enhance personal comfort while reducing energy consumption [8–11]. Passive radiative cooling (PRC) shows outstanding application potential in PTM because it allows 40%–60% of the body heat to be released through radiation [12–14]. Although various radiative cooling materials for the human body have been developed, most of them focus primarily on outdoor cooling, ignoring the important contribution of indoor cooling to personal comfort [15, 16]. Some studies have shown great potential for indoor cooling, while thorough study remain unexplored [17, 18]. Although previous work has achieved both indoor and outdoor cooling, it has not considered the impact of material structure on improving radiation release in the mid-infrared (MIR) band [19].

The challenge in achieving both indoor and outdoor cooling lies in ensuring excellent sunlight reflection while endowing materials with emission and transmission radiation capabilities. High sunlight reflection performance can be accomplished through multilayer structures [20, 21], porous structures [22, 23] and composite structures [24, 25]. These structures, however, concentrate on the reflection in sunlight band (0.3–2.5 µm) and ignore the reflection in the MIR band (2.5–25 μm) will impede the release of thermal radiation from the human body. To address this limitation, the introduction of a full-scale structure design offers a promising solution. This design features a microscale distribution that is concentrated in the 0.3–2.5 μm band while being nearly negligible in the 2.5–25 μm band. Such a structure effect can be utilized to enhance reflectance within the sunlight band, while simultaneously suppressing reflectance in the MIR band.

It is essential to analyze the mechanisms of thermal radiation release, considering the two modes of thermal radiation from an object (transmission and emission) [26]. PRC materials can be categorized into radiation transmission and radiation emission types [13, 27]. For instance, common radiation transmission material polyethylene (PE) has been confirmed to allow human thermal radiation to transmit through, enabling heat to be directly dissipated into the external environment [28–30]. However, the thickness of PE materials must be restricted to less than 150 µm, which reduces their reflectance of sunlight, making PE more suitable for indoor cooling [31]. Radiation emission materials like polyvinylidene fluoride hexafluoropropylene (PVDF-HFP) contain abundant C–F bonds that facilitate the emission of thermal radiation from the human body into the colder universe, making them ideal for outdoor cooling [32, 33]. Unfortunately, this emission process relies on the atmospheric window as a heat transfer channel [34]. When this channel is absent in indoor environment, cooling performance can be significantly impaired. Therefore, integrating radiation emission and radiation transmission is necessary to achieve indoor and outdoor cooling.

Selective emission fabric, which only emitting radiation within the 8–13 μm band, shows excellent potential in simultaneous indoor and outdoor cooling [35, 36]. The full-scale structure effect of the fabric provides the foundation for ensuring low radiation reflection in the MIR band, while the selective emission characteristics enable the material to emit radiation solely within the 8–13 μm band, thereby facilitating the thermal radiation transmittance across the entire MIR band. Therefore, if a combination of full-scale structure and selective emission capabilities is achieved, radiative cooling capabilities for both indoor and outdoor environments will be realized.

Here, we show a polyvinylidene fluoride–polyvinylpyrrolidone (PVDF-PVP) fabric design with passive radiative cooling that functions both indoors and outdoors. Through the full-scale structure design, the resulting nanometer-scale semi-interpenetrating pores and randomly distributed micrometer-scale fibers distinguished the refractive index mismatch at the polymer–air interface and increased multiple Mie scattering efficiency, achieving a satisfied reflectance of 94% in the sunlight band and a low reflectance of 6% in the MIR band. We fully utilized the selective emission properties of PVDF, endowing the material with 81% thermal radiation emission capability in the atmospheric window band and 25% thermal radiation transmission capability in the MIR band. The synergy between emission and transmission characteristics addresses the limitations of single-function materials, facilitating cooling performance in both indoor and outdoor applications. This work provides an innovative approach for the next generation of more intelligent PTM fabrics.

## Experimental Section

### Materials

PVDF (Mw = 1,000,000) was purchased from Arkema. PVP (Mw = 36,000) was purchased from Aladdin Biochemical Technology Co., LTD. DMF was purchased from Tianjin Fuyu Fine Chemical Co., LTD. All reagents were used directly without further treatment.

### Preparation of Cooling Fabric

Firstly, a certain amount of PVDF and PVP were dissolved in DMF solvent and stirred continuously for 8 h under a 70 °C water bath. Specifically, the mass fraction of PVDF in the solution was 8%, 10%, 12%, respectively. The mass ratio of PVDF and PVP was 2:1, 1:1, 1:2. The obtained PVDF-PVP solution was applied to SS-2535H electrospinning device (Yong Kang Le Ye Co., Beijing, China), using a 17-gauge needle tip with a voltage of 13 kV and a feed rate of 0.5 ml h^−1^. The spinning distance, relative humidity, and ambient temperature during spinning were 15 cm, 40%, and 25 °C, respectively. After this, the obtained PVDF-PVP film was immersed in deionized water and subjected to ultrasonic etching for 3 h to remove the PVP partially. The etched film was freeze-dried to obtain cooling fabric. The sample was named FP-X–Y, where X represented the PVDF content in the precursor solution, Y represented the PVP content, and FP-12-0 represented the pure PVDF fabric.

### Materials Characterization

The morphology of fabric was observed by field emission scanning electron microscopy (SEM). Image J software was used to calculate fiber diameters from SEM images. Brunauer–Emmett–Teller and density functional theory models were used to calculate the specific surface area and pore size distribution, and a bubble point aperture tester measured the holes between the fiber. Fourier infrared spectroscopy (Nicolet iN10, China) was used to measure the chemical characteristics of fabric to explain the changes in functional groups during the etching. The refractive index and extinction coefficient of fabric were measured using the spectral ellipsometer (IR-VASE Mark II M-2000UI, USA). The ultraviolet/visible/near-infrared spectrophotometer equipped with a barium sulfate integrating sphere (UV 3600, Japan) was used to measure the sunlight reflection spectrum of fabric in the band of 0.3–2.5 µm. A Fourier infrared spectrometer with an integrating sphere (Nicolet iS50, USA) was used to measure emission and transmission spectra in the band of 2.5–25 µm. The average reflectance, emittance, and transmittance of fabric were calculated according to Eqs. 1–3, respectively.

1 $$R = \frac{{\mathop \smallint \nolimits_{0.3}^{2.5} R(\lambda )I(\lambda ){\text{d}}\lambda }}{{\mathop \smallint \nolimits_{0.3}^{2.5} I(\lambda ){\text{d}}\lambda }}$$

where *R*(*λ*) is the reflectance of the sample at a given wavelength, which is obtained from the measurement results, and *I*(*λ*) is the AM 1.5G global sunlight intensity spectrum. Generally, *λ* is in the band of 0.3–2.5 µm

2 $$E = \frac{{\mathop \smallint \nolimits_{8}^{13} \varepsilon (T,\lambda )I_{b} (T,\lambda ){\text{d}}\lambda }}{{\mathop \smallint \nolimits_{8}^{13} I_{b} (T,\lambda ){\text{d}}\lambda }}$$

where *ε* (*T*, *λ*) is the emittance of the sample at a given temperature *T* and wavelength *λ*, which is obtained from the measurement results, and *I*_*b*_ (*T*, *λ*) is the emission intensity of the blackbody. In general, *T* = 300 K, *λ* in the band of 8–13 µm.

3 $$T = 1 - \frac{{\mathop \smallint \nolimits_{2.5}^{25} R(\lambda )I(\lambda ){\text{d}}\lambda }}{{\mathop \smallint \nolimits_{2.5}^{25} I(\lambda ){\text{d}}\lambda }} - \frac{{\mathop \smallint \nolimits_{2.5}^{25} \varepsilon (T,\lambda )I_{b} (T,\lambda ){\text{d}}\lambda }}{{\mathop \smallint \nolimits_{2.5}^{25} I_{b} (T,\lambda ){\text{d}}\lambda }}$$

where transmittance* T* = 1 − *R* − *E.*

### Calculation of Thermal Radiation Emission

The calculation of thermal radiation emission of materials was analyzed in detail, and the theoretical values were compared with the actual values to ensure the accuracy of the calculated results. First, according to Planck's blackbody radiation law, the spectral radiant force curve of the blackbody at different wavelengths could be obtained, and the integral of the curve was the spectral radiant force of the blackbody (Eq. S1). The spectral emitted force of the actual object can be obtained by multiplying the spectral radiant force of the blackbody with the emissivity at the corresponding wavelength and then integrating. Therefore, by calculating the spectral radiant force of the blackbody, we could ensure the accuracy of the spectral radiant force of the actual object. We obtained that the actual spectral radiant force of the blackbody in the 8–13 µm was 146.58 W m^−2^ (Fig. S1). According to the Stefan–Boltzmann law (4), the entire spectral radiant force of the blackbody at temperature *T* = 300 K was 459.27 W m^−2^. From the relationship between Planck's law and Stefan–Boltzmann's law, the blackbody radiant force between any two wavelengths *λ*_*1*_ and *λ*_*2*_ could be given as Eq. (5).* F*_*b*_ is a function of *λT*, and the proportion of radiation force in a specific wavelength band could be found and calculated according to the blackbody radiation function table, corresponding to the band of 8–13 µm,* F*_*b(0-λ1)*_ was 0.14025, and *F*_*b(0-λ2)*_ is 0.46240 [37]. Then, the theoretical blackbody radiation force was calculated by Eq. (5), and the theoretical blackbody radiation force was 146.97 W m^−2^. The error between the theoretical radiation force and the actual radiation force was only 0.27%, which showed the accuracy of the calculation.

4 $$E_{b} = \sigma T^{4}$$

5 $$E_{{b(\lambda_{1} - \lambda_{2} )}} = \left[ {F_{{b(0 - \lambda_{2} )}} - F_{{b(0 - \lambda_{1} )}} } \right]E_{b}$$

### Calculation of Net Cooling Power

The detail information could be obtained from Eqs. S2–S5.

### Digital Simulation

Combined with Mie scattering theory, the effect of full-scale structure on the scattering efficiency of fabric was further proved theoretically through simulation (Fig. S2). We used the finite difference time domain (FDTD) method based on Lumbrical 2022 R2 software for analysis and simulation. A full-field scattering field (TFSF) light source was used to simulate the scattering efficiency of size distribution from 0.01 to 2.2 µm. Perfect matched layer (PML) boundary conditions and scattering field monitors were adopted for the simulation.

### Cooling Measurement

In the outdoor cooling tests, an anemometer (VC816, China) was used to record the changes in wind speed during the measurements, a solar power meter (CEL-FZ-A, China) measured the intensity of sunlight radiation, and a temperature recorder (VC8801-16, China) was employed to measure the temperature of the fabric both indoors and outdoors. Additionally, a 20 × 20 cm^2^ heater was used to simulate human skin temperature. The outdoor cooling performance was carried out at Northeast Forestry University in Harbin, China. In order to obtain the actual cooling performance, the fabric was pasted on the protective clothing and infrared thermal images were recorded in indoor and sunny outdoor environments within 30 min.

## Results and Discussion

### Design of Cooling Fabric

Generally, indoor and outdoor cooling, with the premise of minimizing the heat input (high sunlight reflection), is attained by adjusting transmit/reflect/emit thermal radiation. The cooling fabric is designed based on three criteria (Fig. S3): (i) has to possess full-scale structure effect for satisfied sunlight reflection and low MIR radiation reflection; (ii) contains functional groups capable of emitting thermal radiation within the atmospheric window band to achieve outdoor human cooling; and (iii) endows thermal radiation transmission performance in the MIR band to facilitate indoor human cooling.

**Fig. 1:**
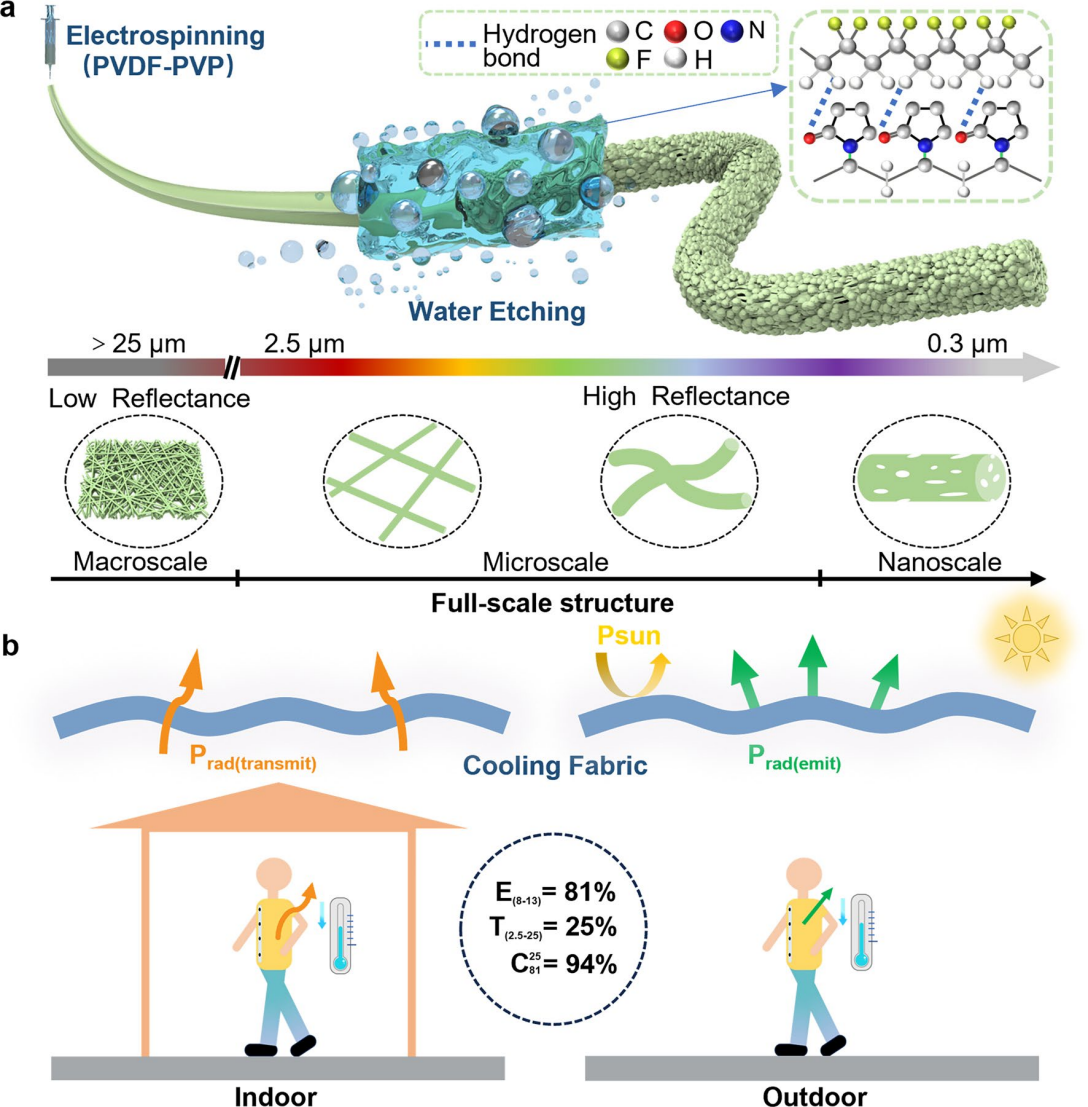
Design and preparation of cooling fabric. **a** Design of full-scale structure cooling fabric. **b** Schematic of indoor and outdoor cooling

Based on these designs, as shown in Fig. 1a, PVDF-PVP cooling fabric was prepared via combining electrospinning technology with ultrasonic water etching [38, 39]. The full-scale structure included nanoscale semi-interpenetrating pores on the fiber surface, microscale fibers, and microscale voids between the fibers, concentrated on the sunlight band, and was distant from the MIR band. The structure effect enhanced the Mie scattering efficiency and reflection ability of the sunlight band while weakening the reflection in the MIR band, which meant that the thermal radiation of the human body readily passed the fabric. PVDF, with C–F bonds, was selected as the matrix material to meet the requirements of selective emission. The absorption and vibration characteristics of the C–F bond provided a strong human thermal radiation emission capability within the atmospheric window band. The low radiation absorption capability within the non-atmospheric window band demonstrated effective transmission performance for human thermal radiation. As a result, this full-scale structure fabric with selective emission characteristic achieved satisfied cooling capability for the human body (Fig. 1b).

### Microstructure of Cooling Fabric

**Fig. 2:**
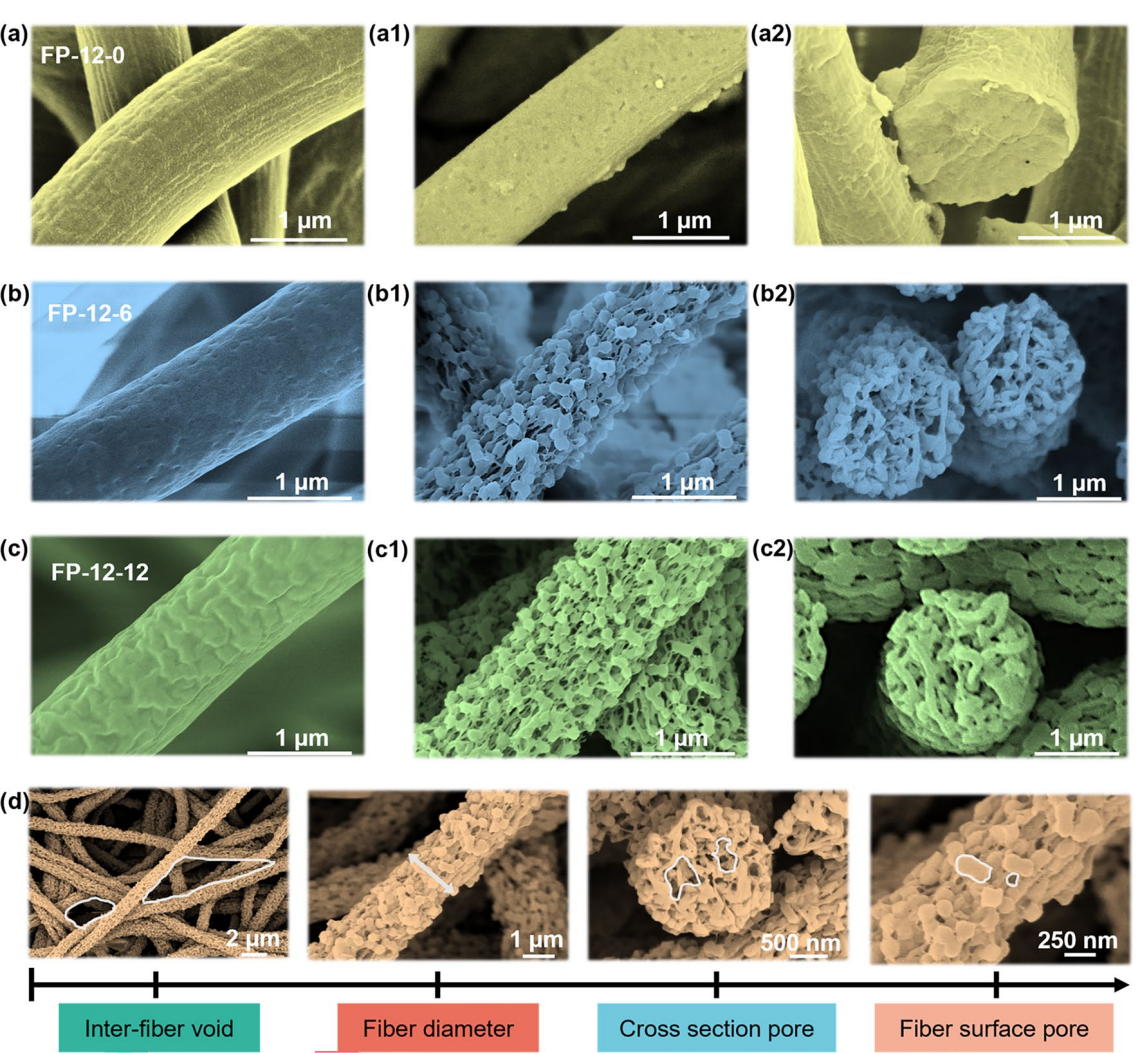
SEM images of the fabrics with different ratios. **a** Morphology of FP-12-0 fiber without etching. **a1, a2** Etched FP-12-0 fiber and section morphology. **b** Morphology of FP-12-6 fiber without etching. **b1, b2** Etched FP-12-6 fiber and section morphology. **c** Morphology of FP-12-12 fiber without etching. **c1, c2** Etched FP-12-12 fiber and section morphology. **d** FP-12-6 full-scale structure distribution

According to the Mie scattering theory, an object strongly reflects sunlight with a wavelength that matches its size [40, 41]. Since sunlight is distributed across the micro- and nanoscales, full-scale structure regulation of the fabric is necessary to achieve satisfied PRC performance. Specifically, water-soluble PVP was fed into PVDF for regulating the diameter distribution of the fiber, and then the targeted pore structure was obtained on the fiber surface by ultrasonic water etching [42]. As shown in Figs. S4 and S5, with the increase of PVDF and PVP content, the fiber diameter increased, and the fiber surface exhibited an evolution from bead-like to smooth and uniform structure [43]. However, excessively high content resulted in cross-linking between fibers (Fig. S6), which were detrimental to controlling fiber size [44]. When the ratio of PVDF to PVP was 12:0, 12:6, 12:12, as shown in Fig. 2a–c, the surface of the unetched fibers exhibited slight wrinkles due to the absorption of water vapor by PVP. The average diameter of unetched FP-12-6 and FP-12-12 fiber was 1.2 and 1.4 µm, both within the band from 0.4 to 1.5 µm. They spanned most of the sunlight band and remained outside the MIR band. In addition, the abundant semi-interpenetrating pores from the fiber surface also contributed to the satisfied sunlight reflectance. After simple ultrasonic water etching, FP-12-0 fiber had no obvious pores (Fig. 2a1, a2). The surface of FP-12-6 and FP-12-12 fibers showed apparent pore structure (Fig. 2b1, c1), and adjacent pores were interconnected to form larger pores. The cross-sectional images of the fibers further showed pore structure in Fig. 2b2, c2. Different from penetrating pores, the semi-interpenetrating pores were indispensable to reflecting the sunlight rather than transmitting it [45]. Water etching had a weak impact on the distribution of fibers, in which the FP-12-6 was the most stable and exhibited the full-scale structure (Figs. S8 and 2d).

### Formation Mechanism of Cooling Fabric

**Fig. 3:**
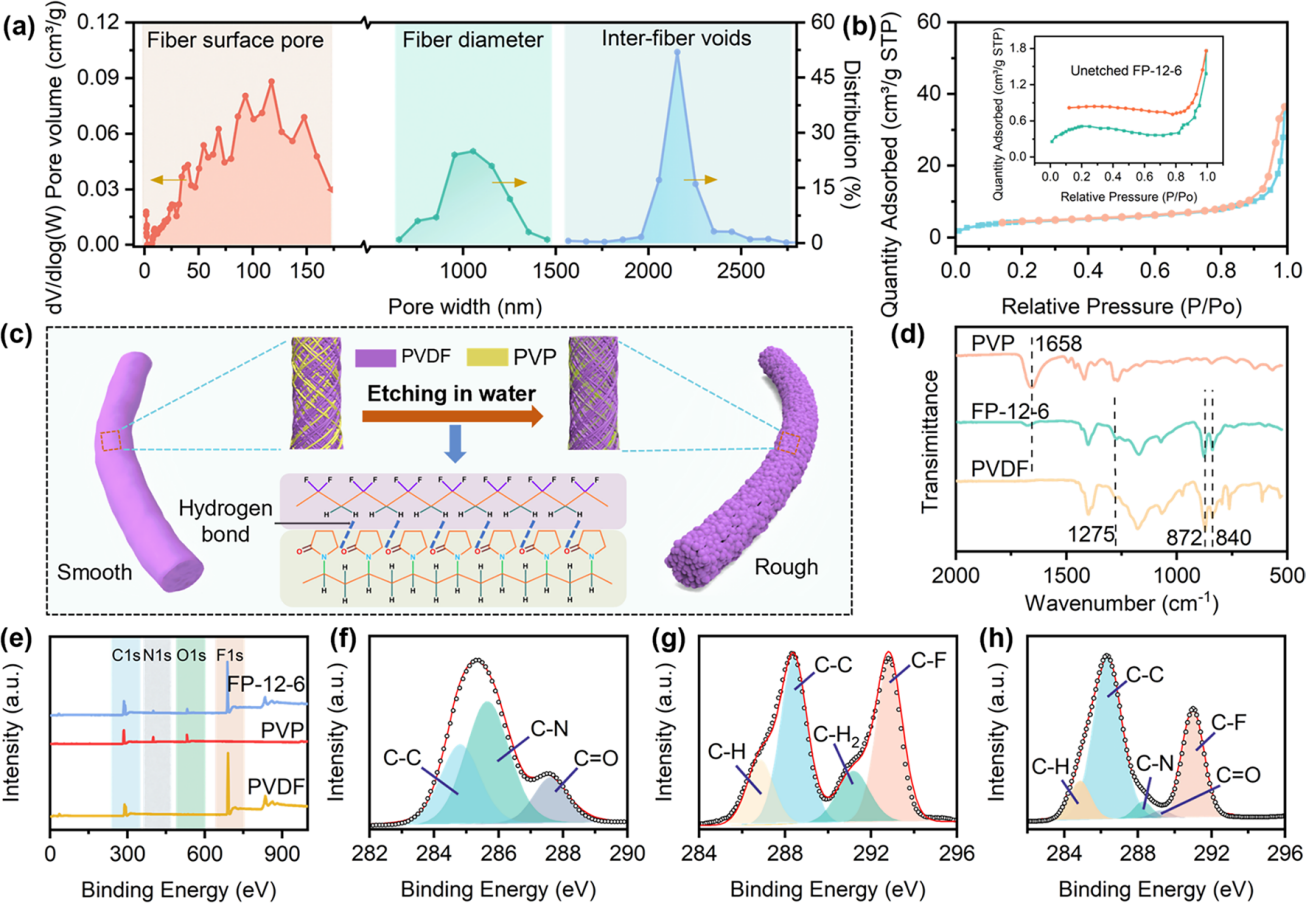
Size distribution and formation mechanism of semi-interpenetrating pores. **a** Size distribution of FP-12-6. **b** Nitrogen adsorption–desorption isotherms curves of FP-12-6 before and after etching. **c** Semi-interpenetrating pores formation mechanism diagram. **d** FTIR image of FP-12-6. **e–h** XPS image of PVP, PVDF and FP-12-6

In view of the above results, a detailed statistical analysis was conducted on the full-scale distribution of FP-12-6 cooling fabric. As illustrated in Fig. 3a, the FP-12-6 fabric showed several dimensional features: the nanoscale semi-interpenetrating pore, the microscale fiber, and the microscale inter-fiber void. The fiber diameter distribution was controlled within the band of 0.6–1.5 µm, endowing the material with the capability to reflect high-wavelength sunlight. Moreover, inter-fiber voids measuring between 1.5 and 2.5 µm covered the shortage to reflect high-wavelength sunlight. The semi-interpenetrating pores mainly consisted of micropores (< 20 nm), mesopores (20–50 nm), and macropores (50–170 nm). A detailed analysis of the semi-interpenetrating pore distribution was performed through nitrogen adsorption–desorption isotherms (Fig. 3b). The type IV curve of the unetched FP-12-6 showed that few macropores were exited. Meanwhile, the type II curve of FP-12-6 indicated that the semi-interpenetrating pores were primarily macropores, which were formed by the interconnection between micropores and mesopores. The macropores provided approximately 94% strong reflection (300–900 nm) for the cooling fabric [46]. Figure 3c shows the formation mechanism of semi-interpenetrating pores. Due to the slight difference in solubility parameters between PVDF (23.2 MPa^1/2^) and PVP (25.6 MPa^1/2^) [47], there was an excellent miscibility between the two components, resulting in their high entanglement within the fibers. In Fig. 3d, pure PVDF and PVP membranes exhibited characteristic peaks of C-F and C=O at 1275 and 1658 cm^−1^, respectively [48]. The C=O group of PVP shifted from 1658 to 1680 cm⁻^1^ in the FP-12-6 fabric, indicating the presence of strong hydrogen bonding interactions between PVP and PVDF. The same phenomenon could also be observed in other samples (Fig. S9). In Fig. 3e, the N 1*s* and O 1*s* peaks in the XPS spectrum of FP-12-6 belong to the unetched PVP. Additionally, in the high-resolution spectra, the binding energy of the C=O group shifted from 287.6 to 289.3 eV (Fig. 3f). The binding energy of C–F decreased from 292.8 to 291.0 eV. These results further confirmed the strong hydrogen bonding interaction between PVDF and PVP. Consequently, during the water etching process, the strong entanglement and hydrogen bonding between the two phases inhibited the etching effect of water. This led to only partial dissolution of PVP in water, resulting in a semi-interpenetrating pore structure in FP-12-6.

### Indoor and Outdoor PRC Performance

**Fig. 4:**
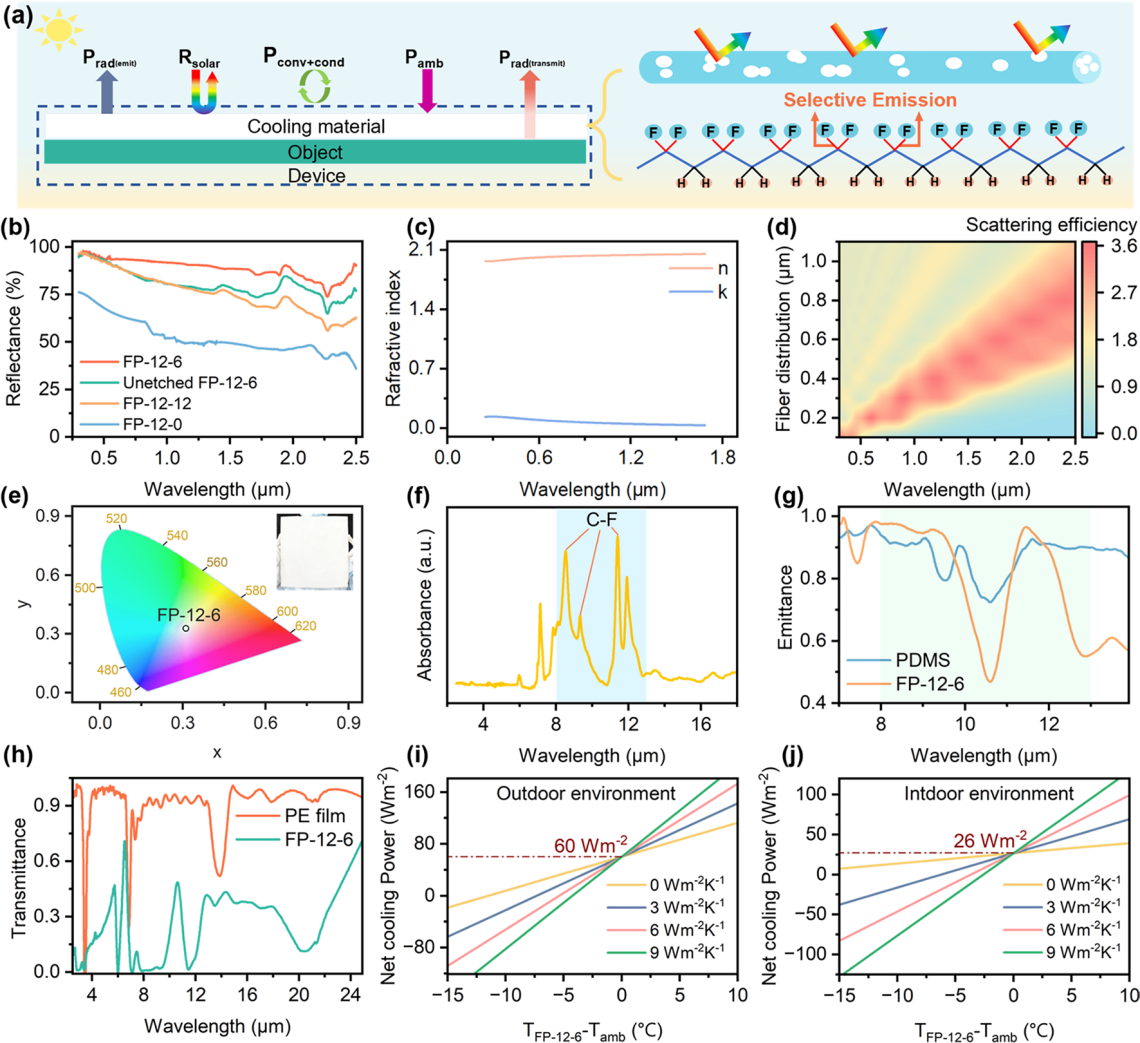
Characterization of sunlight reflection and thermal radiation release performance. **a** Radiation cooling mechanism diagram. **b** Sunlight reflection spectrum of FP-12-6, unetched FP-12-6, FP-12-12, FP-12-0. **c** FP-12-6 refraction coefficient and extinction coefficient. **d** FP-12-6 FDTD simulation. Simulation of fiber size distribution from 0.2 to 1.2 µm. **e** FP-12-6 CIE chromaticity diagram. The illustration is an image of FP-12-6 cooling fabric. **f** FP-12-6 FTIR spectrum. **g** Thermal emission spectrum of PDMS film and FP-12-6. **h** Thermal radiation transmission spectrum of PE film and FP-12-6. **i**, **j** FP-12-6 theoretical outdoor and indoor net cooling power

In general, PRC performance required reducing sunlight absorption while increasing thermal radiation release [49, 50]. Through the full-scale structure and the selective emission regulation, PRC was realized both indoors and outdoors (Fig. 4a). As shown in Fig. 4b, FP-12-6 achieved 94% sunlight reflectance, compared to the state-of-the-art indoor and outdoor cooling materials [19]. In contrast, the unetched FP-12-6 and FP-12-0 displayed weak sunlight reflectance for the absence of the full-scale structure. The uneven full-scale distribution also caused FP-12-12 to exhibit lower sunlight reflectance. Within the sunlight band, the nanoscale semi-interpenetrating pore and microscale fiber distribution endowed the FP-12-6 with satisfied sunlight reflection property. Firstly, the micro-nanostructure featured numerous pore–air interfaces, where light underwent multiple reflections. The cumulative effect of these reflections led to an increase in the total reflectance [49, 51]. According to Fresnel's equations, the mismatch in refractive index further enhanced the reflection effect at the interfaces [52]. FP-12-6 had an average refractive index of 2.0 in the 0.3–1.8 µm band, twice that of air and the near-zero extinction coefficient resulted in a weak absorption of sunlight (Fig. 4c) [53, 54]. Then, Mie scattering theory demonstrated that scattering occurred when the particle size was comparable to the incident wavelength [31, 55]. The micro-nanostructure distribution of FP-12-6 was concentrated within the solar spectrum, which enhanced the Mie scattering effect for sunlight, while having minimal impact on scattering in the MIR band (Fig. S10). FDTD simulation was carried out to further verify the impact of micro-nanosize distribution on scattering efficiency. The scattering efficiency of the 0.01–2.2 µm size distribution was simulated. As shown in Fig. 4d, FP-12-6 strongly scattered sunlight at wavelengths comparable to its fiber from 0.2 to 1.2 µm, which was highly consistent with Mie scattering theory. The semi-interpenetrating pores and inter-fiber voids further contributed to the high scattering efficiency (Figs. S11 and S12). This size distribution covered the entire sunlight band, inducing broadband sunlight scattering. CIE colorimetric analysis indicated that FP-12-6 exhibited a bright white appearance, aligned with satisfied sunlight reflection performance (Fig. 4e) [56].

Imparting specific functional groups in the 8–13 μm, fabrics can be allowed to selectively emit thermal radiation. Based on a careful screening and analysis of the molecular bonds and the corresponding vibrational wavebands of common polymers, we found that PVDF possessed selective emission properties. The main chain of PVDF is composed of C–C bonds, while the side chains contain many C-F bonds. When the FP-12-6 fabric is exposed to sunlight irradiation, the C-F bonds in PVDF undergo significant bending and stretching vibrations due to energy absorption. Simultaneously, electrons transition from higher energy bands to lower energy bands, leading to the emission of photons and heat radiation [57]. As shown in Fig. 4f, the FTIR spectrum of FP-12-6 showed that the C–F bond in PVDF exhibited strong selective radiation emission performance with the 8–13 μm, accompanied by reduced infrared absorption beyond this band, which provided a foundation for achieving radiation emission and radiation transmission. Through infrared reflectance, emissivity, and diffuse reflection tests, the radiation capability of FP-12-6 (220 μm thick) was compared with common radiation-emitting material PDMS film (50 μm thick) and radiation-transmitting material PE film (20 μm thick, Fig. S13). Figure 4g, h shows that PDMS film had 87% radiation emission in the 8–13 µm band, while FP-12-6 reached 81%. PE film achieved 90% radiation transmission between 2.5 and 25 µm, compared to 25% for FP-12-6. The prepared fabric exhibited excellent radiation emission performance compared to typical radiation-emitting materials. Although its radiation transmission was lower, it uniquely combined strong emission capabilities with certain transmission, a rare attribute. Within the band of 2.5–25 µm, the FP-12-6 exhibited a characteristic of extremely low radiation reflection performance, with a reflectance of only 6% (Fig. S14). To more precisely clarify the distinction and relationship between radiative emission and transmission, we defined a new descriptor from the perspective of final cooling, called the radiation cooling ratio: In the MIR band, when the thermal radiation energy is released from the object, the proportion of non-reflected energy to total energy, as well as its distribution. The ratio can be expressed as $C_{E}^{T}$ = *1* − *R*, where *T* is the transmittance in the 2.5–25 µm, *E* is the selective emittance in the 8–13 µm, and *R* is the reflectance in the 2.5–25 µm. The FP-12-6 achieved a radiation cooling ratio ${\text{C}}_{{{81}}}^{{{25}}}$ of 94%, indicating that the majority of human thermal radiation can easily pass through the fabric and the selective emittance and transmittance are 81% and 25%, respectively. The theoretical net cooling power (Pnet) was calculated through the radiative cooling model (Eqs. S2–S5). The Pnet was defined when the sample temperature is equal to the ambient temperature. As shown in Fig. 4i, j, the FP-12-6 had maximum theoretical net cooling power of 60 W m^−2^ outdoors and 26 W m^−2^ indoors, showing satisfactory radiative cooling performance. By optimizing the full-scale structure and selective emission characteristics, FP-12-6 not only demonstrated outstanding radiative cooling capabilities but also provided an innovative solution for achieving comfort in both indoor and outdoor environments.

### Actual Indoor and Outdoor Cooling Performance

**Fig. 5:**
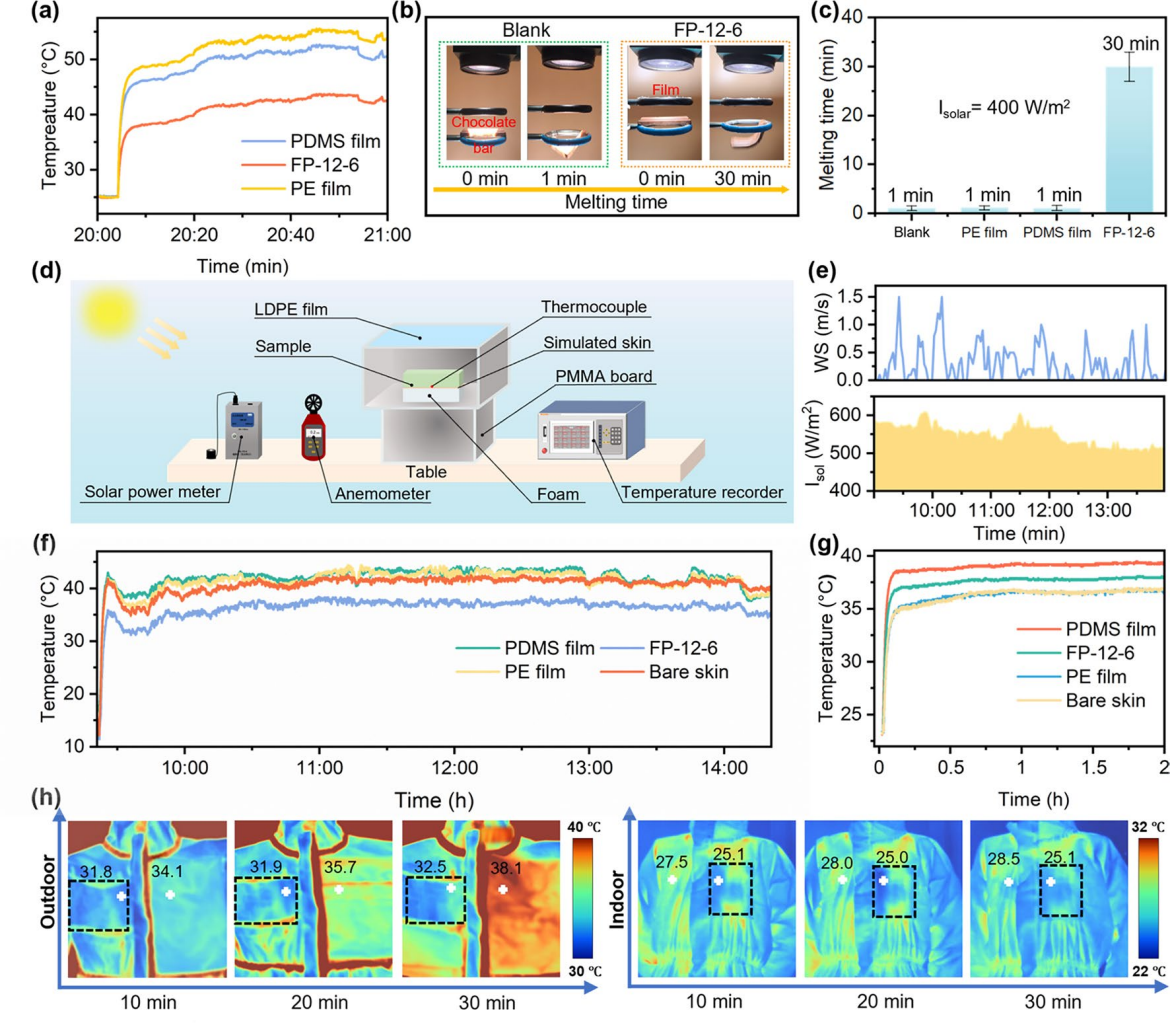
Practical radiative cooling performance. **a** Indoor simulation sunlight test of FP-12-6, PE film and PDMS film. **b** Schematic diagram of chocolate melting test. **c** Results of chocolate melting test. **d** Schematic diagram of outdoor cooling test device. **e** Measurement of wind speed and sunlight intensity of outdoor test. **f** Outdoor cooling test of FP-12-6, PE film and PDMS film. **g** Indoor cooling test of FP-12-6, PE film, and PDMS film. **h** Indoor and outdoor cooling infrared images of FP-12-6 pasted on protective clothing

The indoor and outdoor cooling performance of the FP-12-6 was investigated with bespoke measurement device. To ensure the accuracy of the experimental results, we initially performed simulated sunlight reflection tests. As shown in Fig. 5a. After 1 h of irradiation, the temperatures of the items stabilized, with the PE film, PDMS film, and FP-12-6 reaching 55.1, 52.3, and 43.5 °C, respectively. FP-12-6 exhibited an excellent cooling effect, while the PE film and PDMS film exhibited increased temperatures due to their lower sunlight reflectance (Fig. S15). To better illustrate the sunlight reflectance performance of the cooling fabric, we devised a chocolate melting experiment (Fig. 5b, Videos S1–S4). The experimental setup consisted of a xenon lamp, two circular iron rings, and a support frame. The upper iron ring supported the cooling material, while the lower ring supported the chocolate bar. The xenon lamp intensity was set at 4000 W m^−2^ to accelerate the melting process, and the test results were determined based on the melting time. A blank group without cooling material, a PE film, a PDMS film, and FP-12-6 were tested. As shown in Fig. 5c, the melting time of chocolate was 1 min for the blank group, the PE film, the PDMS film, and 30 min for the FP-12-6. The melting time of chocolate bar covered by FP-12-6 was much longer than the other three groups due to the different sunlight reflection abilities.

Outdoor cooling performance tests were also conducted. Figure 5d shows the plan of the outdoor testing setup, which consisted of an anemometer, a solar power meter, a testing box, a simulated skin (heater), and a temperature recorder. The testing box was made from an uncovered PMMA board topped with a transparent low-density PE film and placed on a Table 1.2 m above the ground to minimize air convection and heat conduction (Fig. S16). Foam boards were placed inside the box to hold the test objects, and thermocouples were set between the test objects and the foam boards. We conducted outdoor temperature measurements of FP-12-6, PE film, PDMS film, simulated skin under direct sunlight on April 7th, 2024, in Harbin, China (126° 38′ 48.5″ E, 45° 43′ 19.6″ N). The simulated skin temperature was set at 37 °C. Environmental conditions were continuously collected during the tests (Fig. 5e), and the temperature changes were continuously monitored for 5 h using temperature recorder. Figure 5f clearly shows that the FP-12-6 exhibited the lowest temperature of all samples under direct sunlight (average 520 W m^−2^ from 9:30 a.m. to 2:30 p.m., with the peak sunlight irradiance of 560 W m^−2^). The temperature of the FP-12-6-covered simulated skin was lower than that of the bare skin and the skin covered by PE and PDMS film by 4.3, 5.0, and 5.5 °C, respectively.

FP-12-6 also exhibited good cooling performance in the indoor environment (Fig. S17). As shown in Fig. 5g, simulating skin temperature at 37 °C, the temperature of the FP-12-6-covered simulated skin was lower than that covered with PDMS film by 1.4 °C, while it was 1.2 °C higher than that covered with bare skin and PE film. The indoor infrared images of the sample also confirmed this conclusion (Fig. S18). The results from both indoor and outdoor cooling measurements demonstrated that the FP-12-6 fabric provided an effective cooling effect for the human body. Additionally, even without a simulated skin, the fabric exhibited well sub-ambient daytime cooling performance under intense sunlight (Fig. S19), highlighting its potential for applications beyond personal thermal management.

The cooling performance of the protective clothing with and without FP-12-6 fabric was assessed indoors and outdoors, with surface temperatures recorded using an infrared camera. As shown in Fig. 5h, in the indoor environments, the temperature of the protective clothing covered by FP-12-6 was lower than the uncovered areas. In sunny outdoor conditions, a significant temperature difference between the two parts of the clothing was also observed. We also tested the wearable performance of FP-12-6, mainly including hydrophobicity, washing resistance, abrasion resistance, mechanical property, moisture permeability, and breathability (Figs. S20 and S21). The test results indicated that the fabric had certain wearable performance and could meet the daily wearing needs of humans. Based on the combined results of indoor and outdoor cooling measurements, the FP-12-6 we prepared effectively cooled the human body, providing a comfortable environment.

## Conclusions

In this study, a full-scale structure cooling fabric with selective emission property was developed. The obtained FP-12-6 performed satisfied sunlight reflectance of 94% and low MIR reflectance of 6% owing to the synergistic scattering effect of the semi-interpenetrating pore and randomly distributed fiber. Moreover, selective emission enabled the FP-12-6 to exhibit 81% radiation emission in the 8–13 µm band and 25% radiation transmission in the 2.5–25 µm band. Theoretical calculations indicated that cooling fabrics can achieve net cooling power of 26 W m^−2^ indoors and 60 W m^−2^ outdoors. Practical results showed that, compared to typical polydimethylsiloxane film, the FP-12-6 demonstrated cooling effects of 5.5 °C in sunlight-rich outdoor environments and 1.4 °C in indoor environments. This work provides a solution to the incompatibility between outdoor and indoor cooling, contributing to the next generation of more intelligent personal thermal management fabric.

## Supplementary Information

Below is the link to the electronic supplementary material.

Supplementary file1 (MP4 1053 KB)

Supplementary file2 (MP4 1317 KB)

Supplementary file3 (MP4 1137 KB)

Supplementary file4 (MP4 1114 KB)

Supplementary file5 (DOCX 6656 KB)
