# Supplementary material for: Selective Emission Fabric for Indoor and Outdoor Passive Radiative Cooling in Personal Thermal Management
Source: Nanomicro Lett. 2025 Mar 19;17:192. doi: 10.1007/s40820-025-01713-4 (PMC11920469; doi:10.1007/s40820-025-01713-4)
Supplement: Supplementary file 5 — Supplementary file5 (DOCX 6656 KB) [file 40820_2025_1713_MOESM5_ESM.docx]

Supporting Information for

**Selective Emission Fabric for Indoor and Outdoor Passive Radiative Cooling in Personal Thermal Management**

Haijiao Yu^1^, Jiqing Lu^1^, Jie Yan^1^, Tian Bai^1^, Zhaoxuan Niu^2^, Bin Ye^1^, Wanli Cheng^1,^ *, Dong Wang^1,^ *, Siqi Huan^1,^ *, Guangping Han^1,^ *

^1^ Key Laboratory of Bio-based Material Science & Technology (Northeast Forestry University), Ministry of Education, Harbin 150040, People’s Republic of China

^2^ Department of Astronautical Science and Mechanics, Harbin Institute of Technology (HIT), Harbin 150001, People’s Republic of China

*Corresponding authors. E-mail: [nefucwl@nefu.edu.cn](mailto:nefucwl@nefu.edu.cn) (Wanli Cheng); [dong.wang@nefu.edu.cn](mailto:dong.wang@nefu.edu.cn) (Dong Wang); [siqi.huan@nefu.edu.cn](mailto:siqi.huan@nefu.edu.cn) (Siqi Huan); [guangping.han@nefu.edu.cn](mailto:guangping.han@nefu.edu.cn) (Guangping Han)

**S1 Planck's blackbody radiation law**

$\text{E}_{\text{bλ}}\text{=}\frac{\text{C}_{\text{1}}\text{λ}^{\text{-5}}}{\text{e}^{{\text{C}_{\text{2}}}/\text{λT}}\text{-1}}$ (S1)

Where *E_bλ_* is the Blackbody spectral radiance, *λ* is the wavelength, *T* is the Thermodynamic temperature, *e* is the base of natural logarithm, *C*_1_ is the First radiation constant, 3.7419×10^-16^, *C*_2_ is the Second radiation constant, 1.4388×10^-2^.

**S2 Net cooling power calculation**

During the day, the net cooling power of a material is the combined results of solar radiation, material thermal radiation, atmospheric thermal radiation, and non-radiative heat exchange. The calculation details are given by the following **Eq. S2**:

$\text{P}_{\text{net}}\text{=}\text{P}_{\text{rad}}\text{-}\text{P}_{\text{atm}}\text{-}\text{P}_{\text{solar}}\text{-}\text{P}_{\text{cond+conv}}$ (S2)

Where *P_net_* is the net cooling power, *P_rad_* is the radiation release capacity of the material, *P_atm_* and *P_solar_* are the heat absorbed by atmospheric and solar radiation, respectively. *P_cond+conv_* is the total power loss of all non-radiative heat transfer processes.

*P_rad_* represents the thermal radiation ability released by the sample, which can be expressed as **Eq. S3**:

$\text{P}_{\text{rad}}\text{=}\int\cos\text{θdΩ}\int_{\text{0}}^{\text{∞}} \text{I}_{\text{BB}}\left( \text{T,}\text{λ} \right)\text{ ε}\left( \text{λ,θ} \right)\text{dλ}$ (S3)

Where $\text{I}_{\text{BB}}\text{=}\frac{\text{2}\text{h}\text{c}^{\text{2}}}{\text{λ}^{\text{5}}}\frac{\text{1}}{\text{e}^{\text{hc/(λ}\text{K}_{\text{B}}\text{T)}}\text{-1}}$is the spectral radiance of a blackbody at temperature *T*. *h* is Planck’s constant, *K_B_* is the Boltzmann constant, and *c* is the speed of light. ∫*𝑑Ω* is the angular integral over a hemisphere, which can be expressed as $\int\text{dΩ=}\text{2}\text{π}\int_{\text{0}}^{\frac{\text{π}}{\text{2}}} \sin\text{θdθ}$. For outdoor environment, both radiation transmission and radiation emission can achieve cooling, therefore 1-*R* is used as the $\text{ε}\text{(}\text{λ}\text{,}\text{θ}\text{)}$ to calculate the cooling power. For indoor environment, as the influence of sunlight in indoor environment is negligible, $\text{ε}\text{(}\text{λ}\text{,}\text{θ}\text{)}$ should be the transmittance of the material at wavelength *λ.*

*P_atm_* is the absorbed atmospheric thermal radiation, which can be expressed as **Eq. S4**:

$\text{P}_{\text{atm}}\left( \text{T}_{\text{amb}} \right)\text{=}\int\cos\text{θdΩ}\int_{\text{0}}^{\text{∞}} \text{I}_{\text{BB}}\left( \text{T}_{\text{amb}}\text{,}\text{ε}\left( \text{λ,θ} \right) \right)\text{ ε}\left( \text{λ,θ} \right)\text{ε}_{\text{atm}}\left( \text{λ,θ} \right)\text{dλ}$ (S4)

Where $\text{ε}_{\text{atm}}\left( \text{λ}\text{,}\text{θ} \right)\text{=}\text{1-}\text{τ}\text{(λ)}^{\text{1}\text{/}\cos\text{θ }}$is the angle-dependent emissivity of the atmosphere. *τ*(*λ*) is the atmospheric transmittance in the zenith direction*.* We used modtran software to simulate the atmospheric transmittance in Mid-Latitude Winter. *T_amb_* was fixed at 300 K.

*P_cond+conv_* is the non-radiative heat transfer power including convection and conduction, which can be expressed as **Eq. S5**:

$\text{P}_{\text{cond+conv}}\text{=}\text{hc}\left( \text{T}_{\text{amb}}\text{-T} \right)$ (S5)

Where nonradiative heat transfer coefficients (*h_c_*) are chosen as 0, 3, 6, and 9 Wm^-2^ K^-1^.

**S3 Supplementary Figures**

**

**

**Fig. S1** Spectral radiation curves of blackbody at different wavelengths


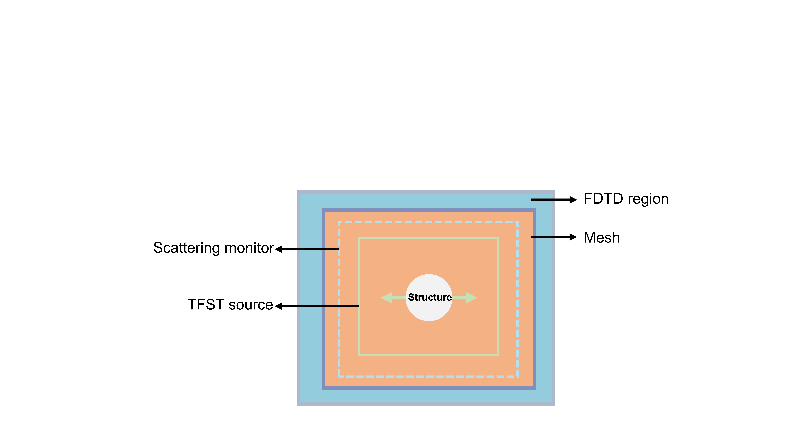


**Fig. S2** The two-dimensional model for Finite-Difference Time-Domain (FDTD)


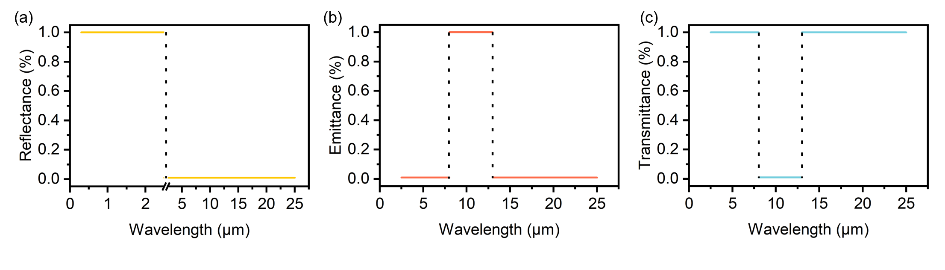


**Fig. S3** Indoor and outdoor cooling spectra under theoretical conditions. **a** Spectrum of reflection. **b** Spectrum of selective emission. **c** Spectrum of transmission


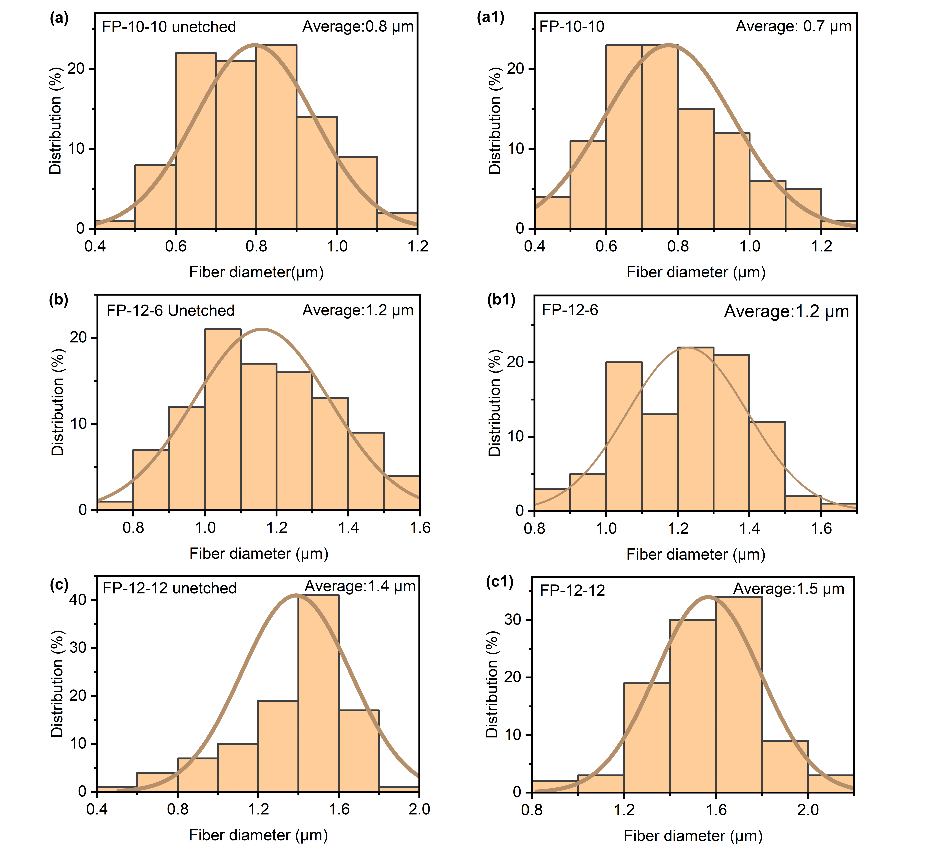


**Fig. S4** Diameter distribution of unetched and etched fiber. **a** unetched FP-10-10. **a1** FP-10-10. **b** unetched FP-12-6. **b1** FP-12-12. **c** unetched FP-12-12. **c1** FP-12-12


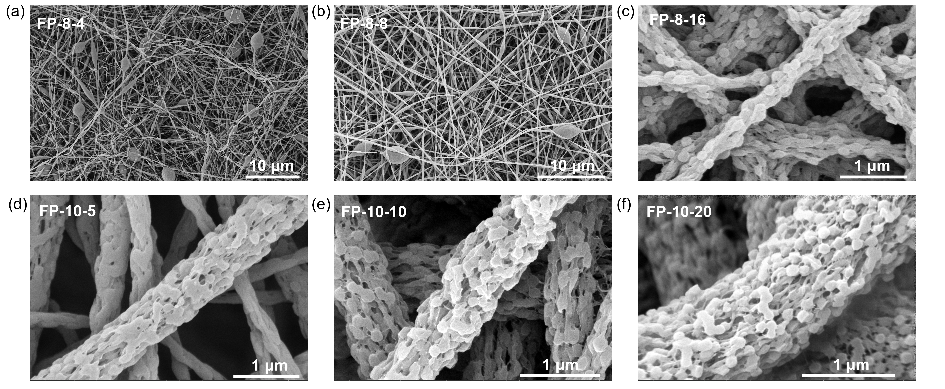


**Fig. S5** SEM image. **a** FP-8-4. **b** FP-8-8. **c** FP-8-16. **d** FP-10-5. **e** FP-10-10. **f** FP-10-20

In this work, the concentration of the precursor solution is determined by the content of PVDF and the ratio between PVDF and PVP, and because the molecular weight of PVDF is much larger than PVP, the influence of PVDF on the concentration of the precursor solution is greater than PVP. When the current ratio of the solution is PVDF: PVP=8:4 and 8:8, excessively low viscosity can cause instability in the jet process, leading to the formation of bead structures. When the current ratio of the solution is PVDF: PVP=8:16, 10:5, 10:10, the fiber size changes significantly and the morphology is unstable after etching. Although FP-10-20 has a relatively stable morphology, the film shrinks severely before and after etching (Fig. S7). Therefore, these concentration ratios are abandoned.

**
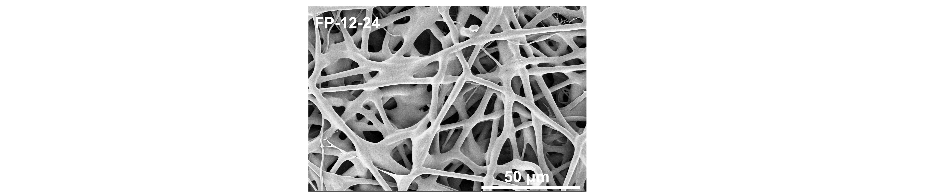
**

**Fig. S6** FP-12-24 SEM images

When the current ratio of the solution is PVDF: PVP=12:24, as the viscosity of the solution further increases, it is difficult for the solution to form a continuous jet from the nozzle. As shown in the Fig. S6, severe cross-linking occurs between fibers, losing their proper fiber structure and making them unusable.


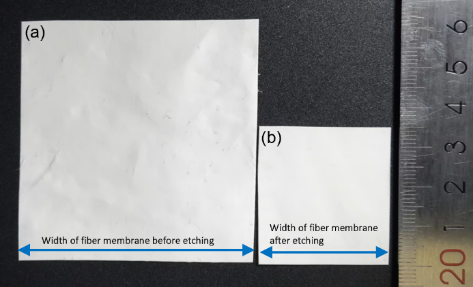


**Fig. S7** Image of FP-10-20 fabric. **a** unetched FP-10-20. **b** FP-10-20. The FP-10-20 underwent significant wrinkling after etching


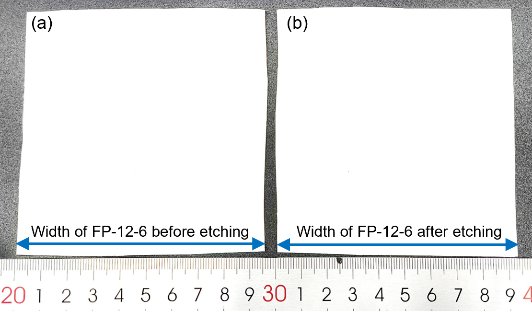


**Fig. S8** Photographs of the FP-12-6 fabric before and after etching. **a** FP-12-6 before etching, **b** FP-12-6 after etching


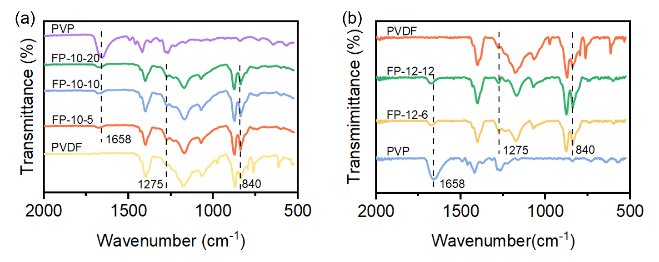


**Fig. S9** FTIR of different samples. **a** FTIR of FP-10-5, FP-10-10, FP-12-20. **b** FTIR of FP-12-6, FP-12-12

**

**

**Fig. S10** Sunlight reflectance and thermal radiation reflectance spectrum of FP-12-6


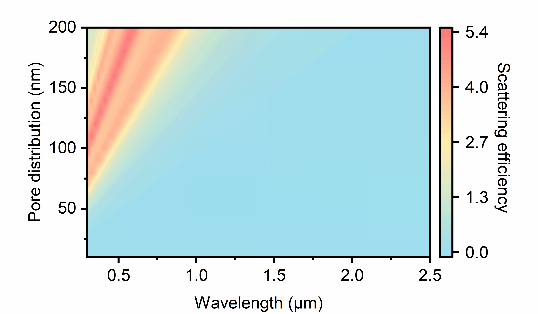


**Fig. S11** Scattering efficiency of FP-12-6 with a pore distribution of 0.01-0.2 μm

**

**

**Fig. S12** Scattering efficiency of FP-12-6 with a size distribution of 1.1-2.2 μm


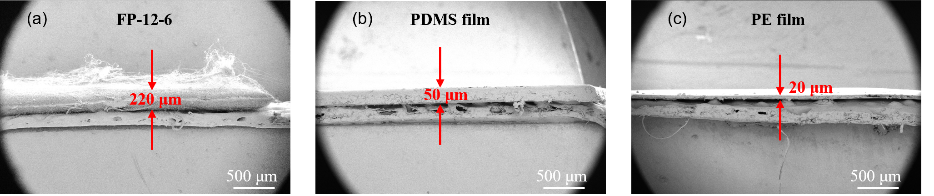


**Fig. S13** Thickness images. **a** FP-2-6. **b** PDMS film. **c** PE film


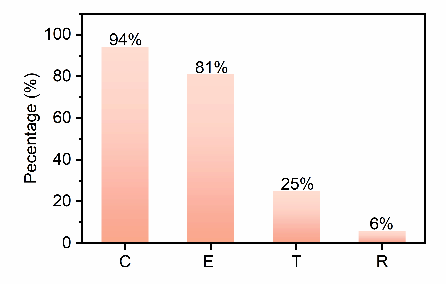


**Fig. S14** Radiation cooling ratio of FP-12-6


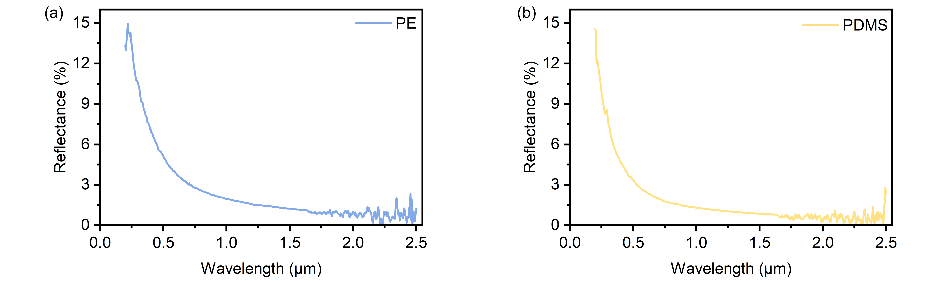


**Fig. S15** Reflectance of PE and PDMS films. **a** PE film. **b** PDMS film


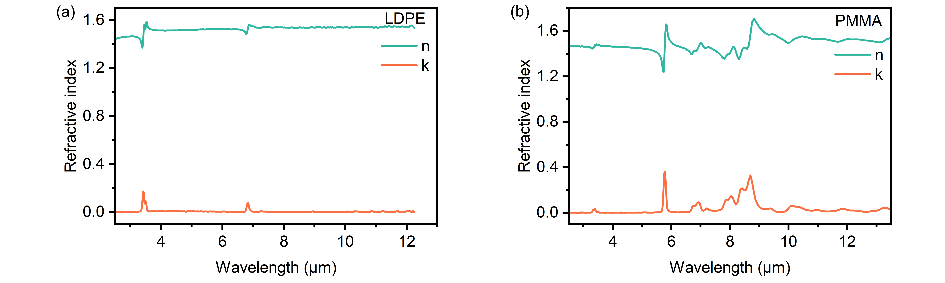


**Fig. S16** Refractive index and extinction coefficient. **a** LDPE film. **b** PMMA board


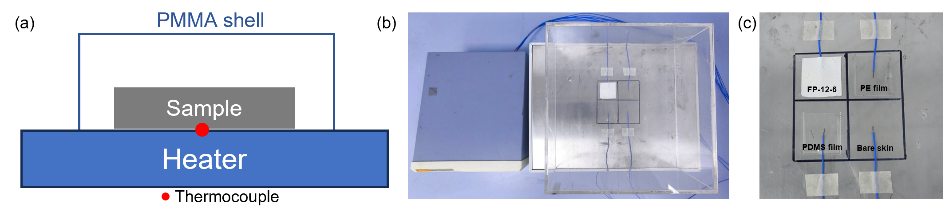


**Fig. S17** Indoor testing schematic diagram. **a** Schematic diagram of indoor detection device. The PMMA shell was used to prevent heat conduction and convection from the surrounding environment. **b** Physical picture of indoor detection device. **c** Enlarged image of indoor detection device


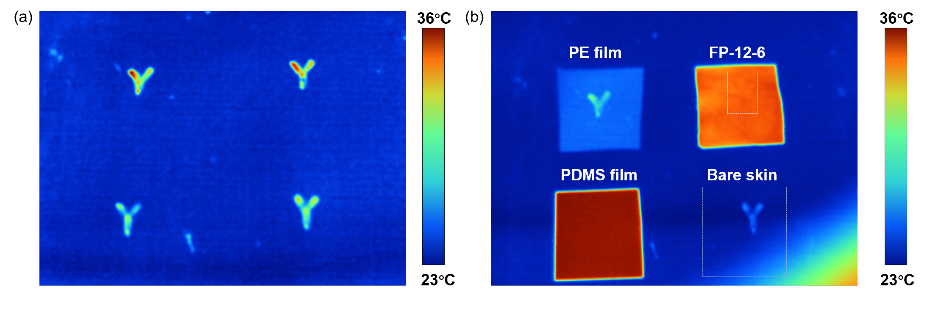


**Fig. S18** Indoor detection infrared images

We took infrared images to evaluate indoor cooling performance. To more clearly demonstrate thermal radiation transmittance, we first marked a "Y" pattern on the bare skin (Heater) (Fig. S18a), then cover the corresponding areas with PE film, FP-12-6, and PDMS film. After a period of time, infrared images were taken. As shown in Fig. S18b, the "Y" pattern was observable on the PE film, indicating its outstanding radiative transmittance performance. Although the "Y" pattern on FP-12-6 was less distinct compared to the PE film, it was still observable, demonstrating that FP-12-6 possessed certain radiative transmittance capabilities. The PDMS film, due to its high radiative emissivity, did not display the "Y" pattern. Based on these tests, the fabricated FP-12-6 fabric exhibited a certain degree of indoor cooling capability.

**
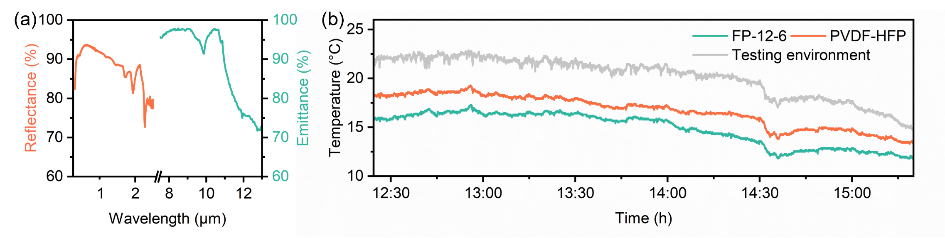
**

**Fig. S19** Outdoor cooling testing without simulated skin. **a** Solar reflectance and atmospheric window thermal radiation emissivity of 12 wt% PVDF-HFP. **b** Outdoor cooling performance of FP-12-6 and PVDF-HFP, experiment conducted on October 16, 2024

An 18 wt% PVDF-HFP fabric had been prepared, achieving 91% solar reflectance and 85% thermal radiation emissivity (Fig. S19a). Outdoor cooling tests comparing FP-12-6 and PVDF-HFP fabrics were then conducted. As shown in Fig. S19b, both fabrics exhibited excellent radiative cooling performance, with FP-12-6 cooling approximately 1.9°C more than PVDF-HFP. This indicated that the materials prepared still achieve good cooling effects even when compared to samples with high reflectance and emissivity.


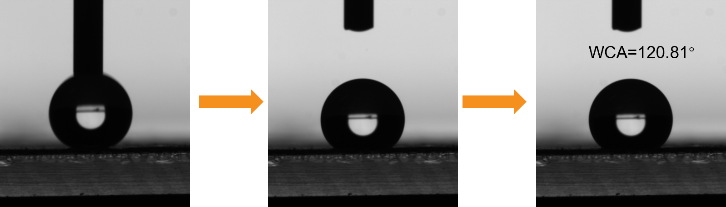


**Fig. S20** Water contact angle of FP-12-6

**
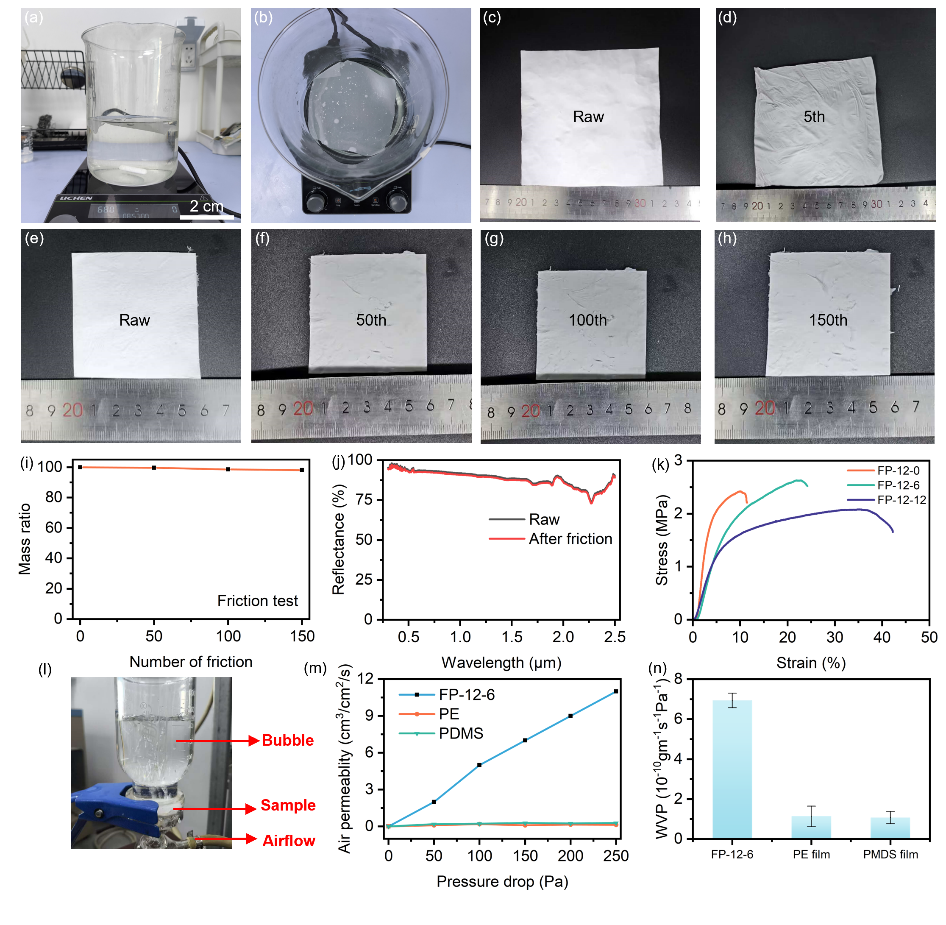
**

**Fig. S21** Wearable performance characterization. **a-d** Washing resistance of FP-12-6. **e-j** abrasion resistance of FP-12-6. **k** Mechanical property of FP-12-0, FP-12-6 and FP-12-12. **l-m** Breathability of FP-12-6, PE and PDMS film. **n** Water vapor permeability of FP-12-6, PE and PDMS film

As shown in Fig. 21a-d, after 5 washes, the fabric exhibited some shrinkage, which was a common phenomenon for most electrospun textiles. As shown in Fig. S21e-j, after 150 cycles of friction testing, no visible damage was observed on the surface of the FP-12-6 fabric, and there was almost no mass loss. The solar reflectance of the fabric remained essentially unchanged before and after the friction test. Therefore, FP-12-6 demonstrated satisfactory abrasion resistance.

As shown in Fig. S21k. Compared to FP-12-0, the mechanical properties of FP-12-6 were improved, while those of FP-12-12 were reduced. The change in mechanical properties was primarily attributed to the alteration in the fiber surface structure. After the water etching treatment, a certain porous structure was formed on the surface of FP-12-6 fibers, which increased the contact area between fibers and provides more physical contact points. These additional contact points not only enhanced the friction between fibers but also promoted mechanical interlocking through physical entanglement, thus improving the mechanical performance. In contrast, for the FP-12-12 fabric, as the PVP content increased, the number of pores on the fiber surface also increased after water etching, damaging the integrity of the fibers and leading to a decrease in mechanical performance.

As shown in Fig. S21l, when FP-12-6 fabric was placed between water and air, continuous air bubbles penetrated without damaging the fabric, demonstrating good air permeability. Quantitative air permeability testing in Fig. S21m further showed that FP-12-6 exhibits air permeability similar to commodity cotton (5 cm³/cm²/s at 100 Pa), which was attributed to its fluffy fiber structure.

As shown in Fig. S21n, due to the dense surface structure of PE and PDMS films, their water vapor permeability was relatively poor. In contrast, FP-12-6 fabric showed a high WVT (7 × 10⁻¹⁰ g·m⁻²·s⁻¹·Pa⁻¹), which was due to the enhanced water vapor permeability provided by the nano and microporous channels.

**Table S1** Previous studies on indoor and outdoor cooling through emission or transmission thermal radiation

| **Material** | **Emission (%)** | **Transmission (%)** | **R_solar_**  **(%)** | **Outdoor cooling** | **Indoor cooling** | **Refs.** |
| --- | --- | --- | --- | --- | --- | --- |
| POM | 73.5 | 48.5 | 94.6 | √ | √ | [S1] |
| PVDF-PVP | 81 | 25 | 94 | √ | √ | This work |
| ZnO-PE | / | 80 | 90 | / | √ | [S2] |
| PVDF-HFP | 94 | / | 96 | √ | / | [S3] |
| HfO_2_-SiO_2_ | / | / | 97 | √ | / | [S4] |
| PMMA-BaSO_4_ | 96 | / | 94 | √ | / | [S5] |
| PVDF-TEOS | 96 | / | 97 | √ | / | [S6] |
| POM-PTFE | 83.2 | / | 95.4 | √ | / | [S7] |
| PT@PEO | 94.8 | / | 95.4 | √ | / | [S8] |
| h-BN | 96.5 | / | 97.3 | √ | / | [S9] |
| PVDF-HFP-EG-FA | 93.4 | / | 95.9 | √ | / | [S10] |

**Table S2** Physicochemical properties of as-spun solutions.

| Spinning solution | Conductivity  (μs•cm^-1^) | Viscosity  (mPa•s) | Surface tension  (mN•m^-1^) |
| --- | --- | --- | --- |
| FP-12-0 | 4.51 | 9900 | 46.5 |
| FP-12-6 | 6.71 | 13885 | 66.9 |
| FP-12-12 | 7.98 | 17350 | 73.8 |
| FP-10-5 | 5.32 | 12575 | 56.4 |
| FP-10-10 | 7.23 | 14434 | 62.7 |
| FP-10-20 | 8.95 | 19545 | 79.3 |

Note: The data in the table is the average value measured at 25℃

**Supplementary References**

1. X.K. Wu, J.L. Li, Q.Y. Jiang, W.S. Zhang, B.S. Wang et al., An all-weather radiative human body cooling textile. Nat. Sustain. **6**, 12 (2023). <https://doi.org/10.1038/s41893-023-01200-x>
2. L.L. Cai, A.Y. Song, W. Li, P.C. Hsu, D.C. Lin et al., Spectrally selective nanocomposite textile for outdoor personal cooling. Adv. Mater. **30**, 7 (2018). <https://doi.org/10.1002/adma.201802152>
3. X.-L. Liao, D.-X. Sun, S. Cao, N. Zhang, T. Huang et al., Freely switchable super-hydrophobicity and super-hydrophilicity of sponge-like poly(vinylidene fluoride) porous fibers for highly efficient oil/water separation. J. Hazard. Mater. **416**, 125926 (2021). <https://doi.org/10.1016/j.jhazmat.2021.125926>
4. A.P. Raman, M. Abou Anoma, L.X. Zhu, E. Rephaeli, S.H. Fan, Passive radiative cooling below ambient air temperature under direct sunlight. Nature **515**, 540 (2014). <https://doi.org/10.1038/nature13883>
5. S.Q. Wang, M.Q. Wu, H. Han, R.X. Du, Z.C. Zhao et al., Regulating cold energy from the universe by bifunctional phase change materials for sustainable cooling .Adv. Energy Mater. **14**, 2402667 (2024). <https://doi.org/10.1002/aenm.202402667>
6. X. Wang, X.H. Liu, Z.Y. Li, H.W. Zhang, Z.W. Yang et al., Scalable flexible hybrid membranes with photonic structures for daytime radiative cooling. Adv. Funct. Mater. **30**, 9 (2020). <https://doi.org/10.1002/adfm.201907562>
7. X.K. Wu, J.L. Li, F. Xie, X.E. Wu, S.M. Zhao et al., A dual-selective thermal emitter with enhanced subambient radiative cooling performance. Nat. Commun. **15**, 815 (2024). <https://doi.org/10.1038/s41467-024-45095-4>
8. P.C. Yao, Z.P. Chen, T.J. Liu, X.B. Liao, Z.W. Yang et al., Spider-silk-inspired nanocomposite polymers for durable daytime radiative cooling. Adv. Mater. **34**, 8 (2022). <https://doi.org/10.1002/adma.202208236>
9. W.M. Tang, Y.H. Zhan, J.R. Yang, X. Meng, X.Y. Zhu et al., cascaded heteroporous nanocomposites for thermo-adaptive passive radiation cooling. Adv. Mater. **36**, 2310923 (2024). <https://doi.org/10.1002/adma.202310923>
10. M.L. Qin, K.H. Jia, A. Usman, S.H. Han, F. Xiong et al., High-efficiency thermal-shock resistance enabled by radiative cooling and latent heat storage. Adv. Mater. **36**, 10 (2024). <https://doi.org/10.1002/adma.202314130>
